# Preventive Effect of Cashew-Derived Protein Hydrolysate with High Fiber on Cerebral Ischemia

**DOI:** 10.1155/2017/6135023

**Published:** 2017-12-31

**Authors:** Jintanaporn Wattanathorn, Wipawee Thukham-mee, Supaporn Muchimapura, Panakaporn Wannanon, Terdthai Tong-un, Somsak Tiamkao

**Affiliations:** ^1^Integrative Complementary Alternative Medicine Research and Development Center and Department of Physiology, Faculty of Medicine, Khon Kaen University, Khon Kaen 40002, Thailand; ^2^Department of Internal Medicine, Faculty of Medicine, Khon Kaen University, Khon Kaen 40002, Thailand

## Abstract

This study aimed to determine the protective effect of cashew nut-derived protein hydrolysate with high dietary fiber (AO) in cerebral ischemic rats induced by the occlusion of right middle cerebral artery (Rt.MCAO). Acute toxicity was determined and data showed that LD_50_ of AO > 5000 mg/kg BW. To determine the cerebroprotective effect of AO, male Wistar rats were orally given AO at doses of 2, 10, and 50 mg/kg for 14 days and subjected to Rt.MCAO. Brain infarction volume, neurological score, spatial memory, serum lipid profiles, and C-reactive protein together with the brain oxidative stress status were assessed. All doses of AO significantly decreased brain infarction in cortex, hippocampus, and striatum together with the decreased oxidative stress status. The improvement of spatial memory and serum C-reactive protein were also observed in MCAO rats which received AO at all doses. In addition, the decreased serum cholesterol, TG, and LDL but increased HDL were observed in MCAO rats which received high dose of AO. Taken all together, AO is the potential protectant against cerebral ischemia. The improvement of oxidative stress, inflammation, and dyslipidemia might play roles in the actions. However, further researches are required to understand the precise underlying mechanism.

## 1. Introduction

Currently, ischemic stroke has been regarded as an important cause of mortality and morbidity at all economic levels worldwide [1]. However, it mostly occurs in the elderly people and patient outcomes after stroke are highly influenced by age [2]. Ischemic stroke produces the great impacts on the quality of life of both patients and their families. In addition, it also produces a great burden on the annual healthcare budget. Therefore, it has been regarded as one of the important health problems. Despite its importance, the therapeutic efficacy is still limited. Therefore, the safe alternative therapies for long-term prophylaxis are currently required.

Recently, nutrition has been recognized as an important factor for stroke prevention [3]. Several studies have demonstrated that a porcine-derived small peptide, cerebrolysin, can improve many neurological disorders including cerebral ischemia [4–6]. It can decrease apoptosis induced by oxidative stress [7]. Although it can produce a great benefit, it is expensive. Moreover, many of the elderly especially in Asian countries prefer to consume plant derived peptide. In addition to the small peptide, the high fiber food also decreases the risk of stroke [8]. Based on the multitarget approach concept, the benefit of the small peptide with high fiber on brain ischemia has been considered.

Cashew or* Anacardium occidentale*, a plant in a family of Anacardiaceae, has a great amount of nutrient. The pseudofruit or cashew apple is a rich source of dietary fiber [9] whereas the nut contains abundant protein [10]. Therefore, cashew can be served as the natural resource for developing food supplement targeting at ischemic stroke prevention. Based on the stroke prevention benefit of peptide and dietary fiber mentioned earlier together with the synergistic effect concept, we aimed to develop the cashew-based protein hydrolysate with high fiber (AO), a novel stroke protectant food, and to determine the preventive effect of AO in animal model of ischemic stroke induced by right middle cerebral artery occlusion (Rt.MCAO).

## 2. Materials and Methods

### 2.1. Preparation of Cashew Nut-Derived Protein Hydrolysate Containing High Fiber

Cashew nuts were cleaned in order to remove all foreign matters such as dust, stone, and dirt, cut into small pieces, and soaked in the boiling water for 10 minutes. Then, distilled water was added at a ratio of 1 : 1 (w/v) and adjusted to pH 8. The suspension was stirred and boiled in water bath at 55°C for 30 minutes. The sample was hydrolyzed by adding 2% alcalase enzyme. After 4 hours of hydrolysis, the inactivation process of enzyme was induced by heating at 90°C for 15 minutes in water bath. Then, the mixture was subjected to a 4100-rpm centrifugation for 10 minutes. The supernatant was collected and concentrated by using freeze dryer [11].

In this study, bagasse of cashew was obtained from the waste generated by fruit pulp industrialization and provided by Srisuphaluck Orchid Company, Phuket, Thailand. Cashew apple residue was chopped into small pieces and dissolved in water at a ratio 1 : 1 (w/v). The suspension was adjusted to pH 5, boiled in water bath at 60°C for 10 minutes, and filtered through cheese cloth. The filtrate was immersed in 95% ethanol at room temperature for 24 hours. The mixture was occasionally agitated. This process was repeated twice. Finally, it was dried in oven at 65°C and grinded in to powder and used as a sample of dietary fiber [12]. The yielded percentage was 9.09%.

To obtain the desired doses of the cashew nut derived protein hydrolysate with high fiber (AO), the protein hydrolysate at doses of 1, 10, and 100 mg/kg BW were mixed with dietary fiber at dose of 2 g/kg BW.

### 2.2. DPPH Radical Scavenging Assay

DPPH radical scavenging activity was measured using the method of Cotelle et al. [13] with a slight modification. The total volume of 2.8 ml of tested samples with the concentrations ranging from 5, 10, 25, 50, 100, 250, 500, and 1000 *μ*g/ml was added to each tube which contained 0.2 ml of DPPH (100 *μ*M in methanol), mixed, and incubated at 37°C in the dark condition for 30 minutes. The absorbance at 517 nm was recorded via spectrophotometer. The percentage inhibition of DPPH radical was calculated by comparing the results of the test with those of the control (not treated with extract) using the following equation:

(1) Percentage  inhibition=1−A  sample517 nmA  control517 nm×100.

### 2.3. Ferric Reducing Antioxidant Power (FRAP) Assay

Reducing power of cashew hydrolysate was performed according to method of Benzie and Strain [14] with some modifications. FRAP reagent which contained 25 mL of 300 mM acetate buffer (pH 3.6), 2.5 mL of ferric tripyridyl triazine [Fe(III) (TPTZ)] solution in 40 mM HCl, and 2.5 mL of 20 mM FeCl_3_·6H_2_O solution was freshly prepared. 10 *μ*L of hydrolysate was dissolved in water and was allowed to mix with 1.8 mL of the FRAP solution at 37°C for 10 min. Absorbance of each sample was recorded at 593 nm.

### 2.4. Evaluation of Cyclooxygenase 2 (COX-2) Inhibition Activity

COX-2 activity was measured using the method of Jang et al. [15] with some modifications. COX-2 activity was assessed by using N,N,N′,N′-tetramethyl-p-phenylenediamine (TMPD) as a cosubstrate with arachidonic acid (AA). The assay mixture containing heme (10 *μ*L), COX-2 (10 *μ*L), 100 *μ*M Arachidonic acid (20 *μ*L), 10 *μ*M N,N,N′,N′-tetramethyl-p-phenylenediamine (TMPD), and Tris-HCL buffer pH 8.00 (150 *μ*L) was mixed with 10 *μ*L of various concentrations of sample ranging from 5, 10, 25, 50, 100, 250, 500, and 1000 *μ*g/ml or indomethacin and incubated at 25°C for 30 minutes. Then, the absorbance at 590 nm was measured using microplate reader. The percentage inhibition of COX2 was calculated by comparing the results of the test with those of the control (not treated with extract) using the following equation:

(2) Percentage  inhibition=1−A  sample590 nmA  control590 nm×100.

### 2.5. Experimental Animals

Healthy male and female Wistar rats (300–350 g) from the National Laboratory Animal Center, Salaya, Nakhon Pathom, were used as experimental animals. They were randomly housed 5 per cage, maintained in 12 : 12 light : dark cycle, and given access to food and water ad libitum. The experiments were strictly performed in accordance with the internationally accepted principles for laboratory use and care of the European Community (EEC directive of 1986; 86/609/EEC). The experiment protocols were approved by the Institutional Animal Care and Unit Committee Khon Kaen University, Thailand (Record no. AEKKU 29/2015). All operations were performed under the pentobarbital sodium anesthesia in order to minimize animal suffering.

### 2.6. Experimental Protocols

Acute toxicity was determined according to the protocols described in OECD Guideline 423. In brief, a total of 20 rats (male 10, female 10) were randomly divided into control and treatment groups. The treatment group was orally given the cashew nut-derived protein hydrolysate with high fiber at a single dose of 5000 mg/kg BW while control group received vehicle. The general appearance and behavioral changes were recorded at 1, 2, 4, and 6 hour-observation period after the daily administration for 14 days. All rats were weighed and we observed mortality, behavioral pattern (salivation, fur, lethargy, and sleep), changes in physical appearance, injury, pain, and signs of illness once daily during the 14-day study period. At the end of study, the blood was collected under anesthesia and the hematological and clinical chemistry changes were determined by Srinagarind hospital. After the collection, the principal organs were excised, weighed, and examined both at macroscopic and at microscopic levels. The evaluation at microscopic level was performed via histological study.

In order to determine the cerebroprotective effect of cashew nut-derived protein hydrolysate with high dietary fiber, male Wistar rats (300–350 g) were randomly divided into 7 groups as follows.

*Group I*. Vehicle + sham operation group: animals in this group were orally given vehicle and received sham operation.

*Group II*. Vehicle + MCAO group: all rats were orally given vehicle and subjected to the occlusion of right middle cerebral artery.

*Group III*. Piracetam + MCAO: rats in this group received piracetam, a standard drug claiming for the cerebral blood flow enhancement, at dose of 250 mg/kg BW via oral route, and were exposed to the occlusion of right middle cerebral artery.

*Group IV*. Vitamin C + MCAO: the animals in this group received Vitamin C, a well-known antioxidant, at dose of 250 mg/kg BW via oral route and were exposed to the occlusion of right middle cerebral artery.

*Group V–VII*. AO + MCAO containing high fiber plus MCAO treated groups: rats in these groups were orally given a cashew nut-derived protein hydrolysate with high fiber at doses of 2, 10, and 50 mg/kg BW and subjected to the occlusion of right middle cerebral artery.

The animals in groups II–VII were orally given the assigned substances at a period of 14 days and subjected to the occlusion of right middle cerebral artery (Rt.MCAO), whereas animals in group I were treated with vehicle at the same period and exposed to sham operation. All treated substances were continually administered throughout a 21-day study period. The biochemical assays were performed at the end of study.

### 2.7. Focal Cerebral Ischemic Induction

12 hours prior to the surgery, animals were exposed to food deprivation but were allowed to access water. Then, they were anesthetized by the intraperitoneal injection of thiopental sodium at dose of 60 mg/kg BW. After the anesthetization, the occlusion of right middle cerebral artery was induced in all rats (MCAO) as previously described [16]. In brief, the right common carotid artery and the internal carotid artery were exposed through a neck midline incision. A silicone coated nylon monofilament (4-0) suture was gently inserted into the common carotid artery and up to the internal carotid artery for a distance of 17 mm from the carotid bifurcation. Then, the wound was sutured and the rats were returned to their cages with free access to food and water. The incision sites were infiltrated with 10% povidone-iodine solution for antiseptic postoperative care. Sham operation groups were exposed to the same operational procedures except the suture which was not advanced into the middle cerebral artery.

### 2.8. Evaluation of Brain Infarction Volume

All rats were killed 24 hours after MCAO, and the brain was removed and sectioned at 2-mm thickness. Sections were immersed in 2% TTC (2,3,5-triphenyl tetrazolium chloride) for 30 minutes at 37°C. The staining brain sections were photographed, and the infarction area was determined by measuring the white area of brain section with computer software (Image J 1.4 V).

### 2.9. Behavioral Studies

#### 2.9.1. Neurological Score Assessment

The neurological score was evaluated according to the method of Bederson and coworkers [17]. The grading was performed as described as follows: grade 0: no spontaneous activity, grade 1: spontaneous circling, grade 2: circling if pulled by tail, grade 3: lowered resistance with lateral push without circling, grade 4: contralateral limb flexion, and grade 5: no apparent deficit.

#### 2.9.2. Assessment of Spatial Memory

Spatial memory was evaluated by using Morris water maze test [18]. A circular pull with a diameter of 170 cm was filled with water at 40 cm deep. The top surface was covered with nontoxic powder. The pool was divided into 4 quadrants and the removable platform was immersed at the center of one quadrant. The animal was trained to memorize the location of platform by forming the association between its location and the location of platform by using external cue. The time which the animal spent for finding and climbing on the platform was recorded as escape latency. The immersed platform was removed from its location 24 hours later. The animals were tested again and the mean time spent in the target quadrant in order to search for the missing platform was regarded as the retention time.

### 2.10. Homogenate Preparation

At the end of experiment, homogenate of right hippocampus, cerebral cortex, and striatum were prepared in 1 mL of 0.1 M phosphate buffer, pH 7.4. The obtained brain homogenate was adjusted to 10% w/v and subjected to a 3,000*g*-centrifugation (microcentrifuge SIG 1- 15PK) at 4°C for 15 minutes. The supernatant was harvested and used for further biochemical assessments.

### 2.11. Biochemical Analysis

#### 2.11.1. Malondialdehyde (MDA) Level Assessment

Level of malondialdehyde (MDA) was monitored by using thiobarbituric acid reacting substances (TBARS) assay. In brief, 100 *μ*L of sample was mixed with the solution containing 100 *μ*L of 8.1% (w/v) sodium dodecyl sulphate (Sigma-Aldrich), 750 *μ*L of 20% (v/v) acetic acid (Sigma-Aldrich) (pH 3.5), and 750 *μ*L of 0.8% thiobarbituric acid (TBA) (Sigma-Aldrich). The solution was heated in a water bath at 95°C for one hour and cooled immediately under running tap water. Then, 500 *μ*L of chilled water and 2500 *μ*L of butanol and pyridine (Sigma-Aldrich) [15 : 1 v/v] were added to each tube and mixed thoroughly with vortex (Vortex-Genie 2). Then, the solution was centrifuged at 800 ×g for 20 minutes. The upper layer was harvested and the optical density was measured at 532 nm with spectrophotometer (GENESYS 20). Various concentrations of 1,3,3-tetraethoxypropane (TEP) (Sigma-Aldrich) were used as standard [19]. The level of MDA was expressed as nmol·mg^−1^ protein.

#### 2.11.2. Determination of Scavenging Enzymes Activities

The determination of superoxide dismutase (SOD) was carried out based on the inhibitory effect of SOD on the reduction of nitroblue tetrazolium (NBT) by the superoxide anion generated by the system xanthine/xanthine oxidase as previously described elsewhere [20]. Absorbance was measured using a spectrometer (UV-1601, Shimadzu) at 550 nm and the SOD activity was presented as units per milligram of protein (U mg/protein). One unit of enzyme activity was defined as the quantity of SOD required to inhibit the rate of reduction of cytochrome by 50%. Catalase (CAT) was performed based on the disappearance of H_2_O_2_ in the presence of brain homogenate at 490 nm [21]. The activity of CAT was expressed as *μ*mol H_2_O_2_/min/mg protein. Glutathione peroxidase (GSH-Px) was determined using t-butyl hydroperoxide as substrate [22]. The activity was expressed as U/mg protein. One unit of the enzyme was defined as micromole (*μ*mol) of reduced nicotinamide adenine dinucleotide phosphate (NADPH) oxidized per minute.

### 2.12. Assessment of Serum C-Reactive Protein

Serum C-reactive protein was determined according to the protocol provided by Thermo Scientific with slight modifications. In brief, 100 *μ*L of standard or sample was added per well of the microwell plate. Then, the plate was sealed and incubated overnight at 4°C with gentle shaking. After the incubation, the plate was washed 3 times with 350 *μ*L of wash buffer. Following the final wash, invert and tap the plate on a paper towel to remove residual buffer. Then, 100 *μ*L of diluted HRP-conjugated antibody (1 : 100) was added to each well of the plate, sealed, and incubated for 1 hour with gentle shaking at room temperature. Following the incubation, each well was washed 3 times with 350 *μ*L of wash buffer. Streptavidin solution at the volume of 100 *μ*L was added to the reaction mixture, sealed, and subjected to a 45-minute incubation with gentle shaking at room temperature. The solution was removed and the washing was repeated for 3 times. Following this step, 100 *μ*L of the TMB substrate solution was added to each well. The reaction mixture was exposed to a 30-minute incubation with gentle shaking at room temperature. The reaction was stopped by adding 50 *μ*L of stop solution to each well and the absorbance at 450 nm was recorded.

### 2.13. Blood Analysis

Serum preparation was prepared after blood collection. The separation of serum was performed by using cooling centrifugation at 2500 rounds per minute (rpm) for 10 minutes. All serum hematological and clinical chemistry changes were determined using photometric analyzer at Srinagarind hospital, Faculty of Medicine, Khon Kaen University, Khon Kaen, Thailand.

### 2.14. Statistical Analysis

Data are shown as mean ± standard error of mean (SEM). Statistical significance was set at *p* value < 0.05. Data analysis was performed using ANOVA followed by Tukey post hoc test.

## 3. Results

### 3.1. Biological Properties and Amino Acid Profiles of Cashew-Derived Protein Hydrolysate

The biological activities related to the pathophysiology of cerebral ischemia were assessed to evaluate the cerebroprotective potential of the developed product. It was found that the IC_50_ value of the developed product obtained via DPPH was less than 5 *μ*g/mL while IC_50_ value of ascorbic acid which was used as positive control was 15.05 ± 0.36 *μ*g/mL. Data obtained from FRAP assay showed that IC_50_ of the developed product was 4.90 ± 0.01 *μ*g ascorbic acid/mg sample. In addition to antioxidant effect, anti-inflammation activity was also assessed via the suppression of cyclooxygenase-2 (COX-2). According to the suppression effect of COX-2, it was revealed that IC_50_ value of the indomethacin which served as positive control was 50.94 ± 0.02 *μ*g/mL whereas IC_50_ of the developed product was less than 5 *μ*g/mL. Therefore, our developed product showed high potential of antioxidant and anti-inflammation activities.

The amino acids composition of cashew nut-derived protein hydrolysate in this study was shown in Table 1. Our protein hydrolysate contained abundance of essential amino acids such as lysine, leucine, phenylalanine, and histidine. In addition, it also contained high amount of glutamic acid. Their concentrations were higher than the suggested requirements pattern by FAO/WHO (1990) for adult human. Moreover, the concentration of sulphur containing amino acid such as methionine and cysteine was also very high.

Our data obtained from the amino acid analysis suggested that a cashew nut-derived protein hydrolysate was a high quality food for feeding because it contained abundance of essential amino acids. In addition it also possessed both antioxidant and anti-inflammation activities.

### 3.2. Acute Toxicity of Cashew-Derived Protein Hydrolysate with High Fiber

Our study showed that no abnormal clinical signs, behavioral changes, body weight changes, macroscopic findings, or organ weight changes were observed. It was found that all animals used in this study survived throughout a 14-day study period. The body weight data were shown in Table 2. Both male and female rats showed no significant difference in body weight gain.  Table 3 showed that there were no significant differences in both daily food and water intakes of both male and female rats. The single oral administration of cashew hydrolysate with high fiber at dose of 5000 mg/kg BW also failed to induce the significant organ weight changes as shown in Table 4. In addition, it was found that our data showed no significant changes in hematological and clinical chemistry parameters as shown in Tables 5 and 6.

### 3.3. Protective Effect against Cerebral Ischemia

**Figure 1:**
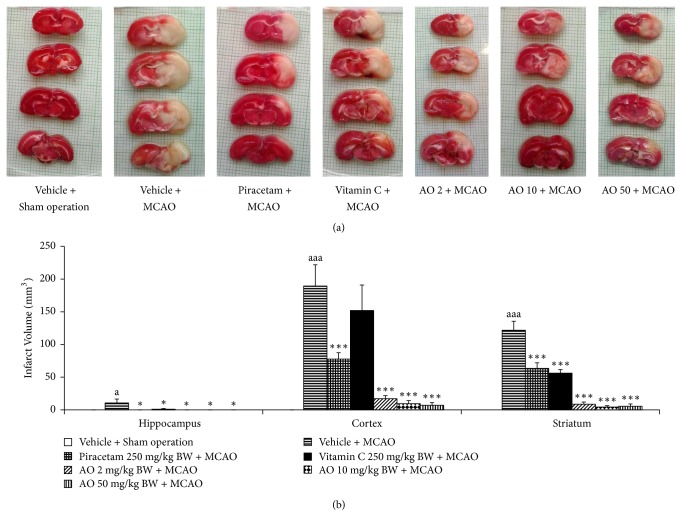
Effect of cashew nut-derived protein hydrolysate with high fiber on brain infarct volume in hippocampus, cerebral cortex, and striatum. (a) Representative photographs of brain infarct volume in various groups assessed by using TTC staining. (b) Volume of brain infarct area, both core and penumbra area of various groups (*N* = 6/group) ^a, aaa^*p* value < .05 and .001, respectively, compared to vehicle + sham operation; ^*∗*, *∗∗∗*^*p* value < .05 and .001, respectively, compared to vehicle + MCAO group.

Figure 1 showed that MCAO induced brain infarction volume in hippocampus, cerebral cortex, and striatum (*p* value < .01, .001, and .001, respectively, compared to vehicle + sham operation group). MCAO rat which received piracetam, a positive control drug used in this study, decreased brain infarction volume in all areas mentioned earlier (*p* value < .05, .001, and .001, respectively, compared to vehicle + MCAO). The significant reduction in brain infarction volumes in hippocampus and striatum of MCAO rats which received vitamin C treatment was also observed (*p* value < .01 and .001, respectively; compared to vehicle + MCAO). Interestingly, all doses of cashew nut-derived protein hydrolysate with high fiber used in this study significantly attenuated the brain infarction volume induced by MCAO in all investigated areas (*p* value < .05 all; *p* value < .001 all and *p* value < .001 all, compared to vehicle + MCAO) as shown in Figure 1.

### 3.4. Effect of Cashew-Derived Protein Hydrolysate with High Fiber on Behaviors

**Figure 2:**
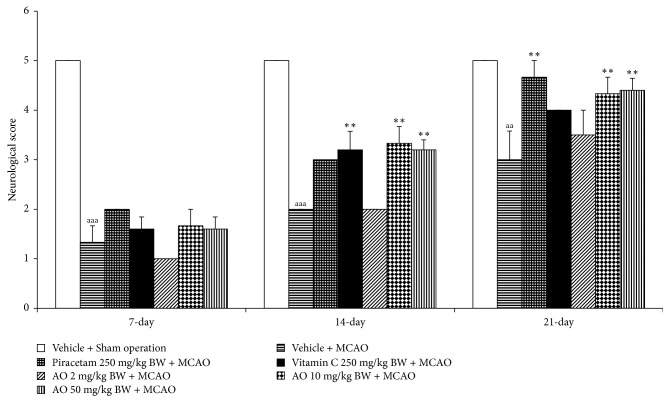
Effect of cashew nut-derived protein hydrolysate with high fiber on neurological score. (*N* = 6/group) ^aa, aaa^*p* value < .01 and .001, respectively, compared to vehicle + sham operation group; ^*∗∗*^*p* value < .01, compared to vehicle + MCAO group.

The effect of cashew-derived protein hydrolysate with high fiber on neurological score was shown in Figure 2. MCAO rat significantly decreased neurological score at 7, 14, and 21 days after the occlusion of middle cerebral artery (*p* value < .001, .001, and .01, respectively, compared to vehicle + sham operation group). MCAO rats which received piracetam significantly increased neurological score at 21 days after treatment (*p* value < .01, compared to vehicle + MCAO group) whereas MCAO rats which received vitamin C showed the significant increase in neurological score at 14 and 21 days after MCAO (*p* value <. 01 all, compared to vehicle + MCAO group). The low dose of cashew nut-derived protein hydrolysate with high fiber failed to improve neurological score while the medium and high doses of the mentioned product significantly improved neurological score (*p* value < .01 all, compared to vehicle + MCAO group).

**Figure 3:**
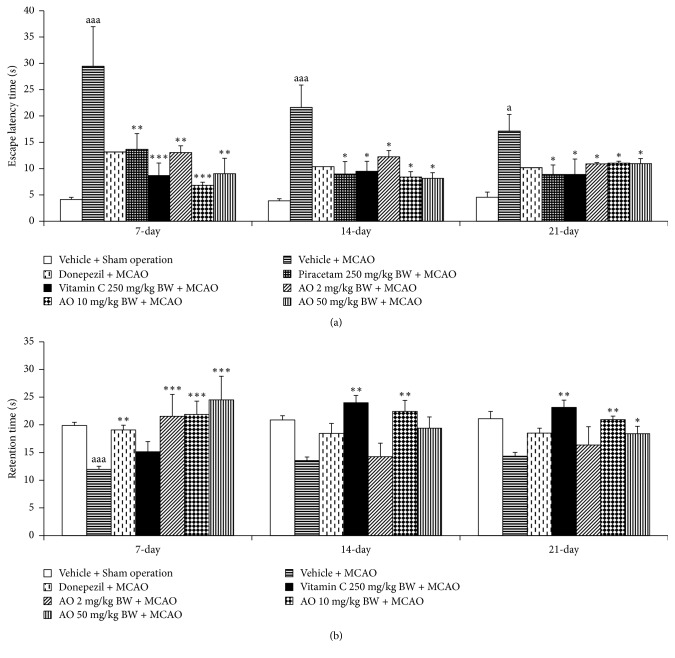
Effect of cashew nut-derived protein hydrolysate with high fiber on spatial memory (a) effect on escape latency, (b) effect on retention time. (*N* = 6/group) ^a, aaa^*p* value < .05 and .001, respectively, compared to vehicle + sham operation group; ^*∗*, *∗∗*, *∗∗∗*^*p* value < .05, .01, and .001, respectively, compared to vehicle + MCAO group.

Figure 3(a) showed that MCAO rats which received vehicle produced a significant increase in escape latency at 7, 14, and 21 days after MCAO (*p* value < .05 all; compared to sham operation group). Piracetam and Vitamin C mitigated the increase in escape latency in MCAO rats at 7, 14, and 21 days after MCAO (*p*-value < .01, .001, and .05 all; .05 all, respectively, compared to MCAO + vehicle). MCAO rats which received cashew-derived protein hydrolysate with high fiber at doses of 1, 10 and 100 mg/kg BW produced the significant reduction in escape latency at 7, 14, and 21 days after MCAO (*p* value < .01, .001, and .01; .05 all; .05 all, respectively, compared to MCAO + vehicle).

The effect of the developed product on retention time was also explored and the results were shown in Figure 3(b). The significant reduction of retention time in MCAO rats was observed at 7 days after MCAO (*p*-value < .001; compared to sham operation). Piracetam enhanced the retention time in MCAO rats at 7 days after MCAO (*p*-value < .01; compared to MCAO + vehicle group) while vitamin C increased retention time in MCAO rats after 14 and 21 days after MCAO (*p*-value < .01 all; compared to MCAO + vehicle group). All doses of the developed product significantly increased retention time at 7 and 21 days after MCAO (*p* value < .001 all; .01, .05, and .05, respectively; compared to MCAO + vehicle group).

### 3.5. Brain Oxidative Stress Status

**Figure 4:**
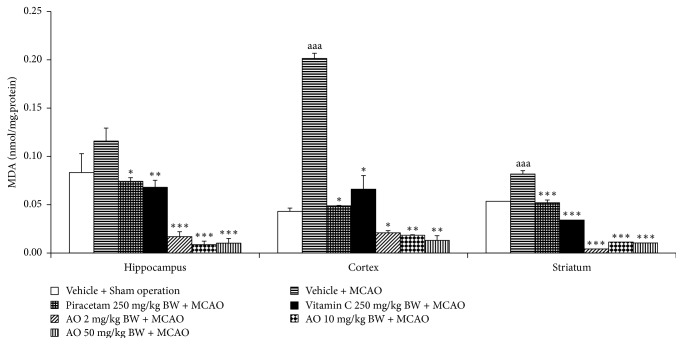
Effect of cashew nut-derived protein hydrolysate with high fiber on malondialdehyde (MDA) level in hippocampus, cerebral cortex and striatum. (*N* = 6/group) ^aaa^*p* value < .001, compared to vehicle + sham operation group; ^*∗*, *∗∗*, *∗∗∗*^*p* value < .05, .01, and .001, respectively, compared to vehicle + MCAO group.

**Figure 5:**
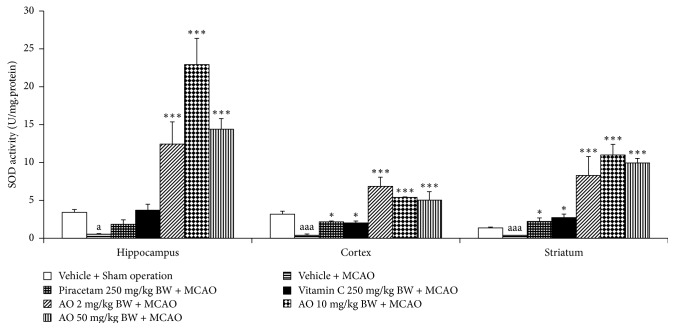
Effect of cashew nut-derived protein hydrolysate with high fiber on superoxide dismutase (SOD) activity in hippocampus, cerebral cortex and striatum. (*N* = 6/group) ^a, aaa^*p* value < .05 and .001, respectively, compared to vehicle + sham operation group; ^*∗*, *∗∗∗*^*p* value < .05 and .001, respectively, compared to vehicle + MCAO group.

**Figure 6:**
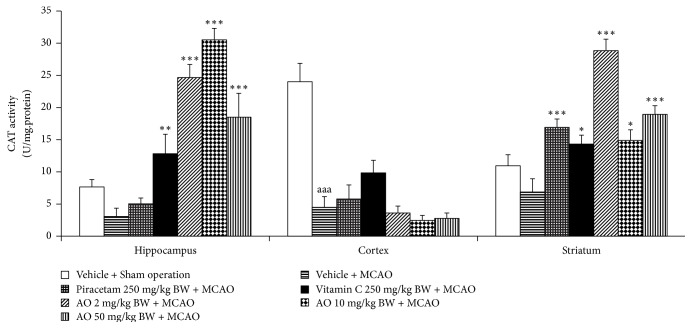
Effect of cashew nut-derived protein hydrolysate with high fiber on catalase (CAT) activity in hippocampus, cerebral cortex and striatum. (*N* = 6/group) ^aaa^*p* value < .001, compared to vehicle + sham operation group; ^*∗*, *∗∗*, *∗∗∗*^*p* value < .05, .01, and 001, respectively, compared to vehicle + MCAO group.

**Figure 7:**
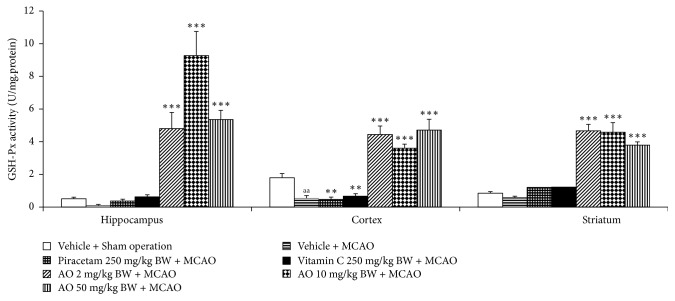
Effect of cashew nut-derived protein hydrolysate with high fiber on glutathione peroxidase (GSH-Px) activity in hippocampus, cerebral cortex, and striatum. (*N* = 6/group) ^aa^*p* value < .01 compared to vehicle + sham operation group; ^*∗∗*, *∗∗∗*^*p* value < .01 and .001, respectively; compared to vehicle + MCAO group.

Figure 4 demonstrated that MCAO produced a significant elevation of MDA level in cerebral cortex and striatum (*p*-value < .001 all; compared to vehicle + sham operation group). Although the elevation of MDA level in hippocampus was also observed but no significant effect was presented. Both piracetam and vitamin C significantly decreased MDA level in hippocampus (*p*-value < .05 and .01, respectively; compared to vehicle + MCAO group), cerebral cortex (*p*-value < .05 all; compared to MCAO rats), and striatum (*p*-value < .001 all; compared to vehicle + MCAO group). MCAO rats which received cashew nut-derived protein hydrolysate with high fiber at doses of 2, 10, and 50 mg·kg/BW produced the significant reduction MDA level in hippocampus (*p*-value < .001 all; compared to MCAO + vehicle group), cerebral cortex (*p*-value < .05, .01 and .01, respectively; compared to vehicle + MCAO group), and striatum (*p*-value < .001 all; compared to vehicle + MCAO group). Since the change of MDA level was under the influence of the enzymatic antioxidant system, we also explored the effect of cashew-derived protein hydrolysate with high fiber on the alterations of main scavenger enzymes including SOD, CAT, and GSH-Px and data were shown in Figures 5–7. In Figure 5, it was found that MCAO significantly decreased SOD activity in cerebral cortex and striatum (*p*-value < .001 all; compared to vehicle + sham operation grop). Both piracetam and vitamin C treatments enhanced SOD activity in cerebral cortex and striatum of MCAO rats (*p*-value < .05 all; compared to MCAO rats). However, cashew nut derived protein hydrolysate with high fiber at all doses used in this study produced the significant elevation of SOD activity in hippocampus, cerebral cortex, and striatum (*p*-value < .001 all; compared to MCAO rats). Figures 6 and 7 showed that MCAO rats significantly decreased CAT and GSH-Px activities only in cerebral cortex (*p*-value < .001 and .01, respectively; compared to vehicle + sham operation group). It was found that piracetam induced the significant reduction in CAT activity only in striatum (*p*-value < .001; compared vehicle + MCAO group) whereas vitamin C produced the significant elevation of CAT activity in both hippocampus and striatum (*p*-value < .01 and .05, respectively; compared to vehicle + MCAO group) as shown in Figure 6. The developed protein hydrolysate at all doses used in this study enhanced CAT activity in hippocampus (*p*-value < .001 all; compared to MCAO rats) and striatum of MCAO rats (*p*-value < .001, .5 and .001, respectively; compared to vehicle + MCAO group).  Figure 7 demonstrated that both piracetam and vitamin C produced the significant increase in GSH-Px activity only in cerebral cortex (*p*-value < .01 all; compared to vehicle + MCAO group) of MCAO rats. Interestingly, the cashew nut-derived protein hydrolysate with high fiber at all doses used in this study significantly enhanced GSH-Px activity in all areas mentioned earlier (*p*-value < .001 all; compared to vehicle + MCAO group).

### 3.6. Inflammatory Marker

**Figure 8:**
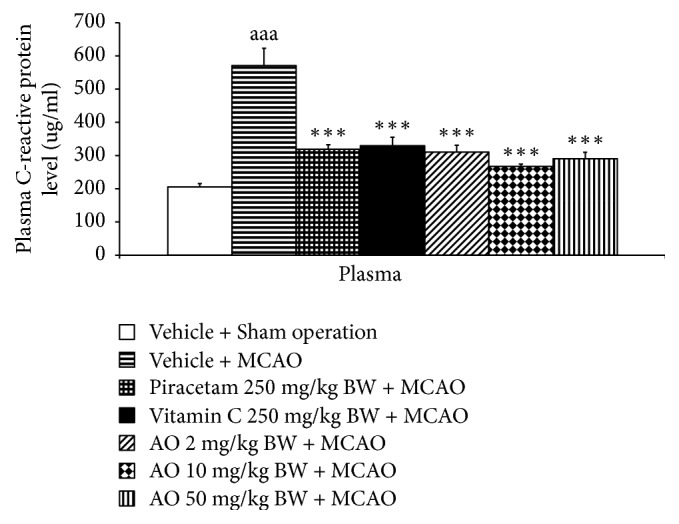
Effect of cashew nut-derived protein hydrolysate with high fiber on serum C-reactive protein level. (*N* = 6/group) ^aaa^*p* value  <.001 compared to vehicle + sham operation group; ^*∗∗∗*^*p* value < .001 compared to vehicle + MCAO group.

Since inflammation plays an essential role on the pathophysiology of cerebral ischemia, we also assessed the effect of the developed product on inflammation by using serum C-reactive protein as index and data were shown in Figure 8. MCAO significantly increased serum C-reactive protein (*p*-value < .001; compared to vehicle + sham operation group). This elevation was attenuated by piracetam, vitamin C, and all doses of the developed product (*p*-value < .001 all; compared to vehicle + MCAO group).

### 3.7. Lipid Profile Changes

Table 7 showed the effect of cashew nut-derived protein hydrolysate with high fiber on lipid profiles. It was found that MCAO rats significantly increased total cholesterol (*p*-value < .01; compared to vehicle + sham operation group). MCAO rats which received cashew nut-derived protein hydrolysate with high fiber at dose of 10 mg/kg BW significantly decreased total cholesterol level (*p*-value < .01; compared vehicle + MCAO group). Interestingly, cashew nut-derived protein hydrolysate with high fiber at dose of 100 mg/kg BW significantly reduced cholesterol, triglyceride, and LDL but increased HDL (*p*-value < .01, .05, .05, and .05, respectively; compared to vehicle + MCAO group).

## 4. Discussion

The present data have demonstrated that single oral administration of cashew hydrolysate with high fiber (AO) at dose of 5000 mg/kg BW produced no effect on mortality, clinical signs, body weight, and organ weight both in male and in female rats throughout the 14 days of study period. Therefore, the cashew hydrolysate with high fiber should be classified in category 5 which is recognized as the lowest toxicity class according to the Guidance Document on Acute Oral Toxicity Testing based on oral LD_50_ value which were recommended by Organization for Economic Cooperation and Development [23–25].

Our in vitro data have clearly demonstrated that AO shows antioxidant and anti-inflammation activities. These effects are also confirmed by our in vivo data which show that AO at all doses used in this study enhance the activities of the main scavenger enzymes including SOD, CAT, and GSH-Px. The elevations of SOD and GSH-Px activities are observed in cerebral cortex, hippocampus, and striatum whereas the enhanced CAT is observed only in hippocampus and striatum. This pointed out CAT activity in cerebral cortex was less sensitive to the treatment. This was in agreement with the previous study which demonstrated that CAT in frontal cortex was less sensitive to pilocarpine treatment [26]. The possible explanation for this phenomenon still required further investigation. The elevation of the scavenger enzymes mentioned earlier may contribute to the important role on the decreased oxidative stress giving rise to the reduction of MDA levels in all brain areas investigated in this study. Since our in vitro data also show that AO also has the capability of decreasing free radicals by itself, the direct antioxidant effect of AO may also contribute a role. Based on these pieces of information, we suggest that the improved brain infarction in AO treated rats may be associated partly with the decreased oxidative stress. Recent study had demonstrated that glutamate and aspartate supplement can alleviate tissue damage induced by oxidative stress [27]. In addition, phenylalanine and sulphur containing amino acids also possess antioxidant activity [28, 29]. Therefore, the antioxidant effect and the effect to enhance scavenging activity of antioxidant enzymes induced by AO may partly relate to the presence of these groups of amino acids in AO.

The current data also clearly demonstrated that the cashew nut-derived protein hydrolysate with high fiber also improved neurological score and spatial memory. Based on the previous information that the improved brain infarction was associated with the improvement of neurological score [30] and spatial memory [31], we suggested that the improved neurological score and spatial memory induced by cashew nut-derived protein hydrolysate with high fiber were also associated with the improved brain infarction. In addition, the elevation of serum C-reactive protein also showed positive correlation with brain infarct volume [32]. Recently, it has been shown that cerebral ischemia can stimulate microglia and leukocyte to release inflammatory cytokine such as interleukin-6 (IL-6) [33] which in turn increase the synthesis and release of C-reactive protein into the circulation [34]. The reduction of serum C-reactive protein in AO treated rats may possibly reflect the reduction of IL-6 which in turn decreases brain damage and infarction [35]. However, this still further investigation. Based on an anti-inflammatory effect of some amino acid such leucine and isoleucine [36], we suggest that the anti-inflammation of AO may be associated with the presence of these amino acids in the hydrolysate.

In this study, we also demonstrated that Rt.MCAO rats also showed the elevation of cholesterol. Interestingly, high dose of AO can decrease cholesterol, LDL, and TG but enhance HDL. Since dyslipidemia plays a vital role in the induction of endothelial dysfunction [37] which plays an important role in the pathogenesis of stroke [38], the improved dyslipidemia of AO may also contribute a role on the improvement of brain damage in cerebral ischemic condition especially at high dose treatment. Based on the reputation of dietary fiber on dyslipidemia and inflammation [39], it is possible that the cerebroprotective effect of AO is associated partly with the effect of dietary fiber content in AO. However, the role of cashew nut- derived protein hydrolysate and the role of the interaction of both cashew nut- derived protein hydrolysate and dietary fiber still cannot be cut off. To provide the precise understanding about the precise underlying mechanisms of the cashew nut-derived protein hydrolysate with high fiber, further researches are essential.

Interestingly, our data showed that AO at all dosage range used in this study showed the protective effect against cerebral ischemia induced by Rt.MACO better than the positive control or piracetam and vitamin C especially in cerebral cortex and striatum. Moreover, AO also improves neurological score, spatial memory, and dyslipidemia better than both substances mentioned earlier. Moreover, it is less toxic. Therefore it is worth further exploring the subchronic toxicity and precise underlying mechanism.

## 5. Conclusions

This study has clearly demonstrated that cashew nut-derived protein hydrolysate with high fiber (AO) is the potential cerebroprotectant against focal cerebral ischemia. The consumption safety is up to 5000 mg/kg BW. Therefore, it is practically safe. Since it can exert the effect on multitargets simultaneously, it may provide high benefit for the complex disorders such as stroke. However, further researches concerning subchronic toxicity and precise underlying mechanisms are required before moving forward to clinical study.

## Figures and Tables

**Table 1:** Amino acid compositions from cashew nut-derived protein hydrolysate.

Amino acid profiles (mg/100 g extract)	Cashew nut
Alanine	918
Arginine	<5.00
Aspartic acid	1266
Cystine	632
Glutamic acid	3055
Glycine	784
Histidine	1823
Hydroxylysine	<5.00
Hydroxyproline	51
Isoleucine	874
Leucine	2229
Lysine	5287
Methionine	387
Phenylalanine	2139
Proline	1011
Serine	674
Threonine	414
Tryptophan	107
Tyrosine	1064
Valine	1347

**Table 2:** Body weight of rats during a 14-day observation period, after a single administration of cashew nut-derived protein hydrolysate with high fiber at dose of 5,000 mg/kg BW via oral route. (*N* = 10/group).

Group	Body weight (g)		Percent change of body weight
	Day 1	Day 14	Day 14
Male			
Control	325.40 ± 1.79	346.20 ± 1.69	6.11 ± 0.004
AO 5,000 mg/kg BW	327.50 ± 1.35	347.80 ± 1.38	6.10 ± 0.003
Female			
Control	227.90 ± 0.72	241.40 ± 0.77	5.91 ± 0.05
AO 5,000 mg/kg BW	234.60 ± 1.35	247.50 ± 1.35	5.67 ± 0.04

**Table 3:** Daily food and water intakes of rats during a 14-day observation period, after a single administration of cashew nut-derived protein hydrolysate with high fiber at dose of 5,000 mg/kg BW via oral route. (*N* = 10/group).

Group	Food intake (g/rat/day)		Water intake (ml/rat/day)	
	Day-1	Day-14	Day-1	Day-14
Male				
Control	22.68 ± 0.19	21.09 ± 4.74	53.40 ± 0.36	56.50 ± 0.11
AO 5000 mg/kg BW	23.18 ± 0.18	21.48 ± 5.40	50.00 ± 0.32	55.00 ± 0.05
Female				
Control	14.24 ± 0.07	14.04 ± 4.74	56.75 ± 0.15	55.25 ± 0.12
AO 5000 mg/kg BW	14.17 ± 0.03	14.76 ± 5.40	55.10 ± 0.19	53.50 ± 0.18

**Table 4:** The weight of various organs of male and female rats after a single administration of a cashew nut-derived protein hydrolysate with high fiber at dose of 5,000 mg/kg BW via oral route. (*N* = 10/group).

Visceral organ (g/kg BW)	Male		Female	
	Control	AO 5000 mg/kg BW	Control	AO 5000 mg/kg BW
Brain	4.12 ± 0.03	3.89 ± 0.05	4.96 ± 0.05	5.09 ± 0.04
lung	4.48 ± 0.14	3.74 ± 0.07	5.57 ± 0.06	5.20 ± 0.02
liver	24.31 ± 0.08	24.96 ± 0.52	37.03 ± 0.38	35.44 ± 0.13
Heart	2.92 ± 0.03	2.81 ± 0.02	4.44 ± 0.04	3.72 ± 0.14
Spleen	2.05 ± 0.03	1.47 ± 0.03	2.95 ± 0.04	2.60 ± 0.03
Pancreas	3.12 ± 0.09	2.36 ± 0.22	4.86 ± 0.14	4.60 ± 0.22
Stomach	4.93 ± 0.07	5.28 ± 0.15	7.10 ± 0.05	6.31 ± 0.07
Intestine	17.99 ± 0.28	19.33 ± 0.22	27.32 ± 0.46	26.04 ± 0.58
Thymus gland	1.07 ± 0.03	0.92 ± 0.03	1.63 ± 0.04	1.76 ± 0.04
Urinary bladder	0.37 ± 0.01	0.51 ± 0.01	0.54 ± 0.01	0.54 ± 0.00
Kidney				
Left	2.78 ± 0.03	2.65 ± 0.03	4.11 ± 0.06	3.67 ± 0.04
Right	2.65 ± 0.05	2.56 ± 0.04	4.13 ± 0.05	3.60 ± 0.03
Adrenal gland				
Left	0.16 ± 0.00	0.19 ± 0.01	0.29 ± 0.02	0.35 ± 0.00
Right	0.13 ± 0.00	0.14 ± 0.01	0.38 ± 0.01	0.42 ± 0.03
Salivary gland				
Left	0.32 ± 0.01	0.29 ± 0.01	0.38 ± 0.01	0.39 ± 0.02
Right	0.33 ± 0.00	0.28 ± 0.01	0.36 ± 0.00	0.26 ± 0.01
Testis/ovary				
Left	3.70 ± 0.03	3.69 ± 0.06	1.64 ± 0.04	1.71 ± 0.06
Right	3.69 ± 0.05	3.66 ± 0.04	1.68 ± 0.04	1.64 ± 0.06

**Table 5:** Hematological parameters of experimental rats after a 14-day observation period following a single administration of cashew nut-derived protein hydrolysate with high fiber at dose of 5,000 mg/kg BW. (*N* = 10/group).

Parameter	Male		Female	
	Control	AO 5000 mg/kg BW	Control	AO 5000 mg/kg BW
Red blood cell (10^6^/ul)	8.45 ± 0.09	8.63 ± 0.11	8.15 ± 0.10	8.72 ± 0.06
Hemoglobin (g/dL)	15.51 ± 0.09	16.02 ± 0.15	14.88 ± 0.17	15.73 ± 0.14
Hematocrit (%)	43.57 ± 0.87	45.60 ± 0.52	43.92 ± 0.63	47.05 ± 0.95
White blood cells (10^3^/ul)	3.49 ± 0.34	2.34 ± 0.19	2.18 ± 0.34	2.01 ± 0.19
Platelet count (10^3^/ul)	656.57 ± 61.59	672.40 ± 20.91	613.00 ± 39.89	713.75 ± 22.59
Neutrophils (%)	29.14 ± 5.46	24.52 ± 1.80	23.02 ± 6.51	21.31 ± 1.75
Lymphocytes (%)	65.43 ± 4.92	67.46 ± 2.23	71.30 ± 7.13	67.50 ± 1.70
Monocytes (%)	3.90 ± 1.14	3.50 ± 0.40	3.40 ± 0.57	4.19 ± 0.52
Eosinophil (%)	0.60 ± 0.15	2.86 ± 0.94	0.96 ± 0.11	5.69 ± 1.92
Basophil (%)	1.00 ± 0.28	0.46 ± 0.21	1.32 ± 0.44	0.56 ± 0.17
Mean corpuscular volume (fL)	55.20 ± 0.50	54.80 ± 0.15	53.84 ± 0.26	51.24 ± 0.59
Mean corpuscular Hemoglobin (pg)	18.26 ± 0.19	17.76 ± 0.06	18.28 ± 0.06	17.75 ± 0.05
Mean corpuscular Hemoglobin concentration (g/dL)	34.30 ± 0.15	35.20 ± 0.33	33.96 ± 0.11	34.71 ± 0.40
Red blood cell distribution width (%)	14.87 ± 0.14	13.86 ± 0.26	14.40 ± 0.13	15.04 ± 0.16

**Table 6:** Clinical chemistry parameters of experimental rats after a 14-day observation period following a single administration of cashew nut-derived protein hydrolysate with high fiber at dose of 5,000 mg/kg BW. (*N* = 10/group).

Parameter	Male		Female	
	Control	AO 5000 mg/kg BW	Control	AO 5000 mg/kg BW
Glucose (mg/dl)	90.40 ± 1.37	89.60 ± 0.73	86.60 ± 1.55	85.40 ± 0.32
Cholesterol (mg/dl)	56.40 ± 0.72	63.60 ± 0.52	60.60 ± 0.69	64.80 ± 0.73
Triglyceride (mg/dl)	72.40 ± 2.24	77.40 ± 0.74	68.80 ± 1.08	68.40 ± 1.15
BUN (mg/dl)	27.51 ± 0.37	22.70 ± 0.09	21.72 ± 0.31	21.42 ± 0.13
Creatinine (mg/dl)	0.40 ± 0.00	0.38 ± 0.00	0.38 ± 0.00	0.42 ± 0.00
ALT (U/L)	38.09 ± 0.74	39.80 ± 0.50	42.34 ± 0.40	47.00 ± 1.33
AST (U/L)	99.40 ± 1.87	124.40 ± 2.40	81.40 ± 1.71	103.80 ± 2.74
ALP (U/L)	29.60 ± 0.68	27.00 ± 0.47	32.60 ± 0.70	30.20 ± 1.07
Total Bilirubin (mg/dl)	0.08 ± 0.00	0.10 ± 0.00	0.10 ± 0.00	0.10 ± 0.00
Sodium (mEq/L)	140.80 ± 0.11	138.40 ± 0.20	140.60 ± 0.12	142.10 ± 0.32
Potassium (mEq/L)	6.72 ± 0.08	6.28 ± 0.09	7.14 ± 0.07	7.07 ± 0.11
Chloride (mEq/L)	97.00 ± 0.13	98.60 ± 0.30	97.40 ± 0.13	97.30 ± 0.24
Bicarbonate (mEq/L)	22.78 ± 0.21	20.20 ± 0.14	21.15 ± 0.10	23.35 ± 0.25

**Table 7:** Effect of various doses of cashew nut-derived protein hydrolysate with high fiber on lipid profiles. (*N* = 6/group) ^aa^*p* value .01, compared to vehicle + sham operation group; ^*∗*, *∗∗*^*p* value .05 and .01, respectively, compared to vehicle + MCAO group.

Group	Parameter			
	Cholesterol (mg/dL)	Triglyceride (mg/dL)	LDL (mg/dL)	HDL (mg/dL)
Vehicle + Sham operation	72.78 ± 4.97	52.31 ± 6.28	14.94 ± 3.39	49.27 ± 4.40
Vehicle + MCAO	92.35 ± 3.55^aa^	72.02 ± 3.09	21.77 ± 0.97	43.27 ± 2.24
Piracetam 250 mg/kg BW + MCAO	88.98 ± 0.32	60.92 ± 2.13	17.29 ± 1.45	45.35 ± 2.54
Vitamin C 250 mg/kg BW + MCAO	84.01 ± 4.62	65.42 ± 3.21	15.23 ± 2.63	50.24 ± 0.54
AO 2 mg/kg BW + MCAO + MCAO	85.17 ± 4.06	70.28 ± 13.42	15.99 ± 1.69	47.36 ± 2.36
AO 10 mg/kg BW + MCAO + MCAO	75.44 ± 4.39^*∗∗*^	56.89 ± 5.81	17.59 ± 3.87	53.45 ± 2.54
AO 50 mg/kg BW + MCAO + MCAO	76.70 ± 2.19^*∗∗*^	46.06 ± 5.18^*∗*^	13.04 ± 2.10^*∗*^	55.19 ± 5.66^*∗*^
